# 3D Quantitative Modeling for Stone Fruit Quality Assessment by LF-NMRI

**DOI:** 10.3390/foods15112012

**Published:** 2026-06-04

**Authors:** Kang Wang, Bing Li, Shan Zeng, Wei Tao, Ke Yang, Zhiguang Yang

**Affiliations:** School of Mathematics and Computer Science, Wuhan Polytechnic University, Wuhan 430023, China; 15871791039@163.com (K.W.); zengshan1981@whpu.edu.cn (S.Z.); taowei@whpu.edu.cn (W.T.); ke.yang@whpu.edu.cn (K.Y.); yangzg@whpu.edu.cn (Z.Y.)

**Keywords:** LF-NMRI, multi-view 3D reconstruction, non-destructive assessment, CVR

## Abstract

The core volume ratio (CVR) is a key indicator for evaluating the proportion of edible fraction in stone fruits. Traditionally, CVR is determined through destructive sampling by separately measuring the masses of the core and entire fruit. Recently, low-field nuclear magnetic resonance imaging (LF-NMRI) has been introduced as a non-destructive alternative, but its sparse sampling limits the ability to achieve accurate spatial and volumetric quantification of fruit quality. To address this limitation, we propose a novel method for high-precision three-dimensional (3D) modeling of stone fruits. The method acquires tomographic LF-NMRI sequences along three orthogonal axes. Each sequence is segmented into pulp and core regions using a SwinUNet deep learning model and converted into point clouds for each view. Point clouds from the three orthogonal views are registered via a genetic algorithm to align structural information from complementary perspectives and fused into a unified 3D model through Poisson surface reconstruction. Using prunes as a representative case, the method enables accurate quantification of core and entire fruit volumes, achieving a CVR estimation with a mean absolute error of 0.13% compared to manual measurements. The proposed three-view reconstruction strategy yields a volumetric error of only 0.73%, significantly outperforming single-view (4.57%) and dual-view (3.73%) approaches. This technology provides a robust and accurate non-destructive solution for 3D internal quality analysis of fruits.

## 1. Introduction

As living standards continue to improve, global fruit consumption has been growing steadily, accompanied by increasing expectations for fruit quality [1]. The fruit industry plays a critical role in the global agricultural economy, with an annual output exceeding one billion tons and showing sustained growth [2]. Fruits are rich in bioactive compounds, including polyphenols, dietary fiber, vitamins, and essential minerals, that contribute significantly to human metabolic regulation and overall health [3]. However, postharvest deterioration has become a growing concern, as internal defects such as decay and pest infestation can occur without visible symptoms. These issues not only compromise fruit quality and shelf life but also pose serious food safety risks to consumers [4].

Traditional fruit quality assessment methods are largely destructive, including physical dissection and chemical analysis. Physical dissection involves cutting open the fruit to inspect internal tissues and identify structural defects. In contrast, chemical analysis quantifies nutritional attributes, such as soluble solids, titratable acidity, and vitamin C, by extracting juice from the pulp [5,6]. Although these methods offer high analytical accuracy, they are time-consuming, labor-intensive, and unsuitable for non-destructive, efficient inspection required in modern industry [7]. In particular, they fail to detect latent internal defects, such as early-stage decay or insect infestation, which often lack external symptoms. Collectively, these limitations underscore the need for non-invasive, efficient, and accurate techniques for internal fruit quality assessment [8].

Early non-destructive methods for fruit quality assessment primarily relied on visible and near-infrared (Vis-NIR) spectroscopy to evaluate chemical composition and physical attributes [9,10]. With advances in technology, hyperspectral imaging (HSI) emerged, combining the rapid analysis capabilities of spectroscopy with the spatial resolution of imaging, enabling quantitative visualization of surface component distribution in fruits [11,12,13]. However, both Vis-NIR and HSI are largely restricted to surface evaluation and lack the penetration depth needed to assess internal features such as moisture distribution, core volume ratio (CVR), and internal defects like decay or pest damage [14]. Traditional CVR measurement still requires fruit dissection and manual separation of the core and entire fruit, which is destructive and unsuitable for large-scale or repeatable assessments [15]. More recently, imaging techniques such as X-ray and computed tomography (CT) have been explored for internal quality evaluation [16,17], but radiation-related safety concerns remain a significant limitation. In contrast, low-field nuclear magnetic resonance imaging (LF-NMRI) offers a safe, non-invasive alternative with high sensitivity to water-rich tissues, showing distinct advantages for internal fruit quality assessment [18].

While LF-NMRI has shown promise for internal fruit quality assessment, its current applications are primarily limited to two-dimensional (2D) imaging. Existing studies have mainly used LF-NMR/LF-NMRI to analyze proton relaxation behavior related to moisture distribution, water mobility, postharvest physiological changes [19], and key physicochemical attributes of fruit tissues. In addition, the integration of LF-NMR with machine learning [20], hyperspectral imaging [21], and image super-resolution techniques [22] has improved the non-destructive detection of bruising, decay, and other quality-related traits. However, these studies are still largely centered on signal analysis, 2D image enhancement, or quality classification, with little attention to non-destructive three-dimensional (3D) reconstruction and quantitative characterization of heterogeneous internal fruit structures. Such 3D analysis remains difficult because sparse data acquisition and low contrast between internal tissues jointly impair segmentation and reconstruction. In particular, ambiguous tissue boundaries reduce segmentation accuracy, and sparsely sampled slices often lead to structural distortion and accumulated errors in 3D volume estimation. Traditional segmentation methods that rely on manual feature extraction are therefore poorly suited to LF-NMRI images with weak contrast and blurred interfaces [23].

To overcome these challenges, this study proposes a multi-view fusion framework for 3D reconstruction of fruit internal structures based on LF-NMRI. Existing LF-NMRI research has primarily addressed 2D analysis, with non-destructive 3D reconstruction under low-contrast and sparse-sampling conditions remaining underexplored. Using prunes as a representative case, the proposed approach enables precise quantification of CVR and demonstrates the potential of LF-NMRI for non-destructive, high-fidelity 3D analysis of internal fruit quality. The main contributions of this study are threefold:

(1) Extension of LF-NMRI application scope from 2D imaging and quality classification to non-destructive 3D reconstruction and quantitative analysis of internal fruit structures.
(2) Improved segmentation of heterogeneous internal tissues through deep learning under weak-contrast and blurred-boundary conditions.
(3) Enhanced 3D reconstruction accuracy from limited slices via multi-view fusion, registration, and surface reconstruction, enabling quantitative evaluation of CVR.

## 2. Materials and Methods

This section provides an overview of the experimental workflow, including sample preparation and experimental design, tomographic data acquisition, image preprocessing, and multi-view 3D reconstruction. Prune samples were scanned using an LF-NMRI system to obtain tomographic sequences along three orthogonal planes. Image preprocessing involved segmentation of core and pulp regions using a SwinUNet-based model, followed by contour extraction of internal and external boundaries. The resulting slice-wise contours were converted into point clouds via coordinate transformation. Point clouds from the three views were registered using a genetic optimization algorithm, and a complete 3D model of the prune and its core was reconstructed through Poisson surface reconstruction.

### 2.1. Sample Preparation

A hierarchical experimental design was employed to evaluate the robustness of the LF-NMRI method. Prune samples were harvested from Kashgar, Xinjiang, China. To account for biological variability, five independent batches were collected at different dates over approximately four weeks during the same commercial harvest season. Inter-batch variation was mainly related to differences in fruit maturity stage and fruit size.

The sample selection criteria required fruits to have a uniform shape, a moderate diameter (35–50 mm), and be free from visible defects such as surface damage, shrinkage, or decay. Only fruits exhibiting an intact epidermis and a natural coating of surface powder were retained to minimize interference from deteriorated tissues during image acquisition and segmentation. Based on these criteria, 10 representative fruits were randomly selected from each batch, yielding a total of N = 50 samples. This design adheres to rigorous statistical standards for characterizing agricultural commodities. All selected prunes were stored at 4 °C to preserve freshness.

### 2.2. LF-NMRI Data Acquisition

**Figure 1:**
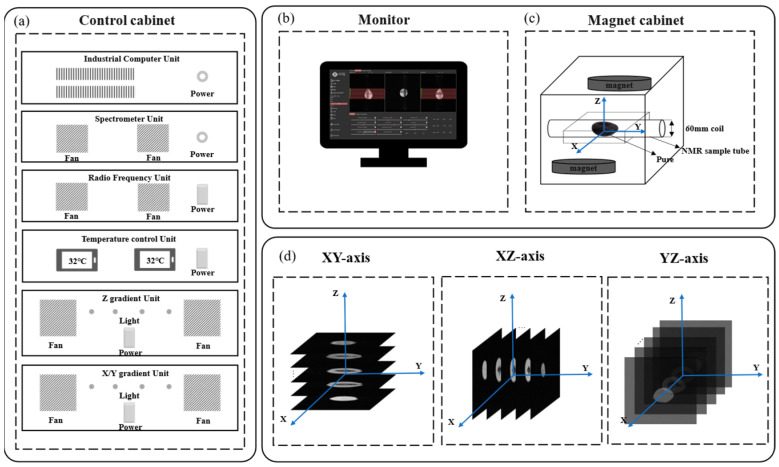
Components of the LF-NMRI system: (**a**) control unit, (**b**) image display monitor, (**c**) magnet cabinet with prune sample, and (**d**) multi-plane slice acquisition setup (XY, XZ, YZ).

Tomographic images were acquired using a LF-NMRI system (NMI20-060H-SO, Niumag Co., Ltd., Suzhou, China) operating at a magnetic field strength of 0.5 T. The LF-NMRI system comprises a control cabinet, a display monitor, a magnet unit, and an orthogonal image acquisition setup (Figure 1). The internal chamber temperature was maintained at 32 °C throughout scanning to ensure magnetic field stability [24]. This temperature corresponds to the manufacturer-specified operating condition for the NMI20-060H-SO system, which is required to stabilize the permanent magnet and associated electronics. Prior to LF-NMRI scanning, samples were equilibrated at room temperature for 2 h to mitigate temperature-induced artifacts [25]. Imaging and preprocessing were conducted using the Niumag NMR Imaging System and the associated Image Processing Software (v3.0).

For the size characteristics of the prune samples, a radiofrequency coil with an inner diameter of 60 mm was selected for imaging. The prune sample was placed at the center of the coil’s inner cavity. First, the T1 and T2 relaxation times of the prune tissue were measured through a preliminary experiment. Based on the measured T1 and T2 values, the parameters of the spin-echo (SE) sequence, specifically the repetition time (TR) and echo time (TE), were optimized. Subsequently, proton density-weighted images (PDWIs) were acquired using the SE sequence. These images clearly distinguish the proton density differences between the pulp and the core, providing a contrast basis for subsequent segmentation. In order to balance image acquisition efficiency with image quality and improve the quality of the obtained point cloud data, the optimized parameters for the SE sequence are: TR = 3000 ms, TE = 18.64 ms, FOV = 150 × 150 mm^2^ with a matrix of (256 × 256) pixels, Slice gap = 0.5 mm, Slice thickness = 1.5 mm, Average = 2, RG (analog gain) = 20 dB, PRG (pre-amplifier gain) = 3 dB, DRG (digital gain) = 5 dB.

To ensure the precise fusion of multi-viewpoint cloud data, it is essential to maintain strict orthogonality of the three imaging planes (XY, XZ, YZ) to obtain accurate spatial structural information. Therefore, during image acquisition, the sample must be firmly fixed in the spatial position within the magnet cavity, as shown in Figure 1d [26]. Based on the sample’s geometric dimensions, the number of images captured for the three orthogonal planes (XZ, YZ, XY) is between 24–26, 17–20, and 15–17, respectively.

### 2.3. Data Preprocessing

To accurately segment pulp and core regions in LF-NMRI images of prunes, a semantic segmentation workflow was constructed by combining preprocessing techniques with a deep learning-based model. Non-local means filtering (smoothing coefficient = 10; window size = 8 × 8 pixels) and pixel-level thresholding were applied to suppress noise and enhance structural contrast while preserving key anatomical details. The denoised images were then fed into an improved SwinUNet network, which integrates the global attention capability of the Swin Transformer with the multi-scale feature representation of U-Net in a symmetric encoder–decoder architecture. This model was selected because LF-NMRI images of prunes present blurred tissue boundaries, heterogeneous intensity distributions, and substantial morphological variability, requiring both global contextual modeling and precise local feature extraction for accurate segmentation.

**Figure 2:**
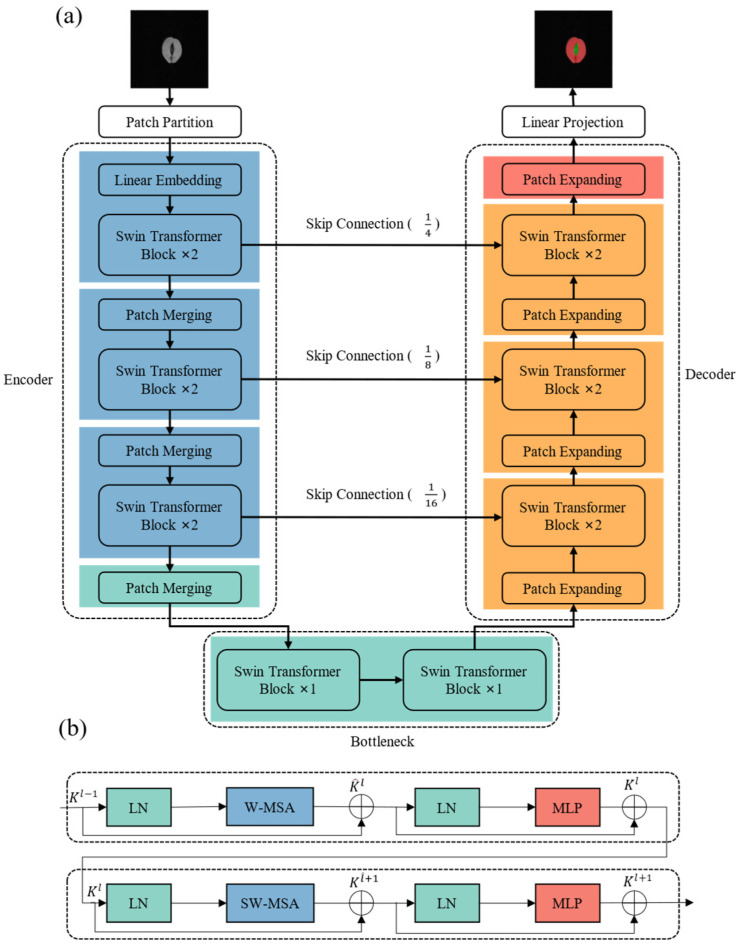
Architecture of the segmentation network: (**a**) Encoder–decoder structure, (**b**) Structure of standard and SW-MSA blocks at the bottleneck.

As shown in Figure 2, the encoder splits the input into non-overlapping 4 × 4 patches and linearly embeds the resulting features through a linear embedding layer, followed by cascaded Swin Transformer blocks with patch merging for hierarchical downsampling. At the bottleneck stage, two consecutively stacked Swin Transformer blocks are used to extract deep semantic features. To better capture long-range dependencies and maintain spatial coherence, the network incorporates both standard and shifted-window multi-head self-attention (SW-MSA) blocks, replacing traditional convolution operations with adaptive attention mechanisms. In the decoding phase (Figure 2a), spatial resolution is progressively restored using patch expansion, with multi-scale features fused via additional Swin Transformer blocks and encoder outputs integrated through skip connections. The final pixel-level segmentation mask is produced through a linear projection layer.

Model training was conducted using an internal LF-NMRI prune dataset constructed in our previous work [27], comprising 1400 manually annotated images covering diverse prune morphologies. The dataset was acquired from prune samples spanning a range of fruit diameters (35–55 mm) and core sizes (core volume: 1.5–3.1 cm^3^), sourced from the same orchard region (Kashgar, Xinjiang, China) across two harvest seasons. Each image was manually annotated by two trained operators with pixel-level labels for three classes: background, pulp, and core. Inter-annotator agreement was verified prior to training. The dataset was randomly split at the fruit level, with all images from each prune sample assigned exclusively to either the training set or the test set. Of these, 1200 images were used for training and 200 for testing. The resulting high-precision segmentation masks enable accurate contour extraction and provide a reliable foundation for the subsequent 3D reconstruction process.

### 2.4. Multi-View 3D Reconstruction

**Figure 3:**
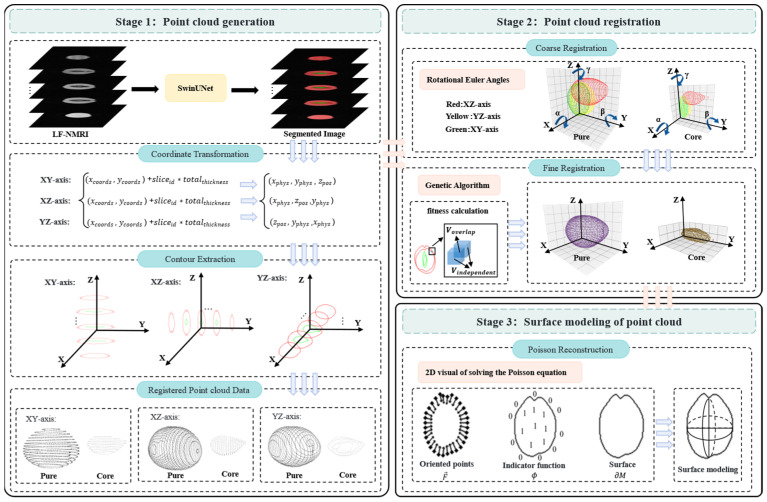
Multi-view 3D reconstruction pipeline: Stage 1: point cloud generation; Stage 2: point cloud registration optimized by genetic algorithm; Stage 3: surface modeling via Poisson reconstruction.

This section presents a three-stage pipeline for 3D reconstruction of prunes based on multi-view LF-NMRI data, including point cloud generation, registration, and surface modeling. As illustrated in Figure 3, the 2D tomographic slices obtained from Section 2.3 are first mapped into 3D physical space via coordinate transformation, enabling spatial consistency between image data and real-world geometry. Next, point clouds from the three orthogonal imaging planes are aligned using a two-step registration strategy comprising coarse alignment and fine registration based on a genetic algorithm. Finally, Poisson surface reconstruction is applied to generate a watertight 3D model that accurately reflects both external morphology and internal structures.

#### 2.4.1. Generation of Point Cloud Data

**Figure 4:**
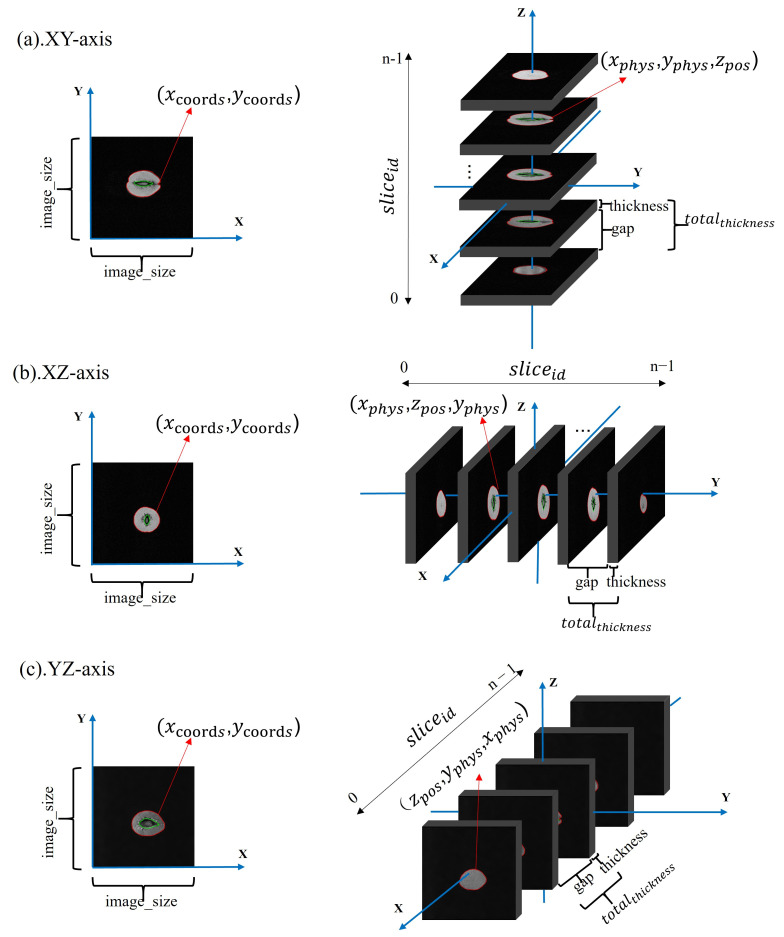
Mapping of 2D slice coordinates to 3D spatial coordinates along: (**a**) XY-axis, (**b**) XZ-axis, and (**c**) YZ-axis.

To generate point cloud data, the pixel coordinates of each 2D slice must be mapped to a unified 3D spatial coordinate system. This requires establishing a quantitative correspondence between image pixels and physical dimensions. As shown in Figure 4, a transformation model is constructed to convert slice-based pixel coordinates into real-world spatial coordinates. This enables accurate geometric mapping from 2D tomographic images to 3D physical space. The coordinate transformation from image space to physical space is defined as follows:

(1) $$\begin{array}{c} x_{phys}=x_{coords}\cdot scale_{factor}-FOV/2\\ y_{phys}=y_{coords}\cdot scale_{factor}-FOV/2\\ z_{pos}=slice_{id}\cdot total_{thickness}-(count\cdot total_{thickness})/2 \end{array}$$

where $x_{phys}$ and $y_{phys}$ are the physical coordinates along the X and Y axes, respectively; $scale_{factor}$ is the conversion ratio between pixels and physical units, calculated as the field of view (FOV) divided by the image size; $total_{thickness}$ denotes the combined thickness and inter-slice gap of a single slice; $slice_{id}$ is the index of the current slice; count is the total number of slices; $z_{pos}$ gives the final physical coordinate along the Z axis.

After coordinate transformation, contour extraction is applied to the segmented images to delineate geometric boundaries required for 3D reconstruction. The findContours algorithm in OpenCV 4.8.0 [28] is used to extract both the outer surface and inner core boundaries with high spatial precision. This topological analysis-based method ensures the structural completeness of both the pulp and core during subsequent point cloud generation.

**Figure 5:**
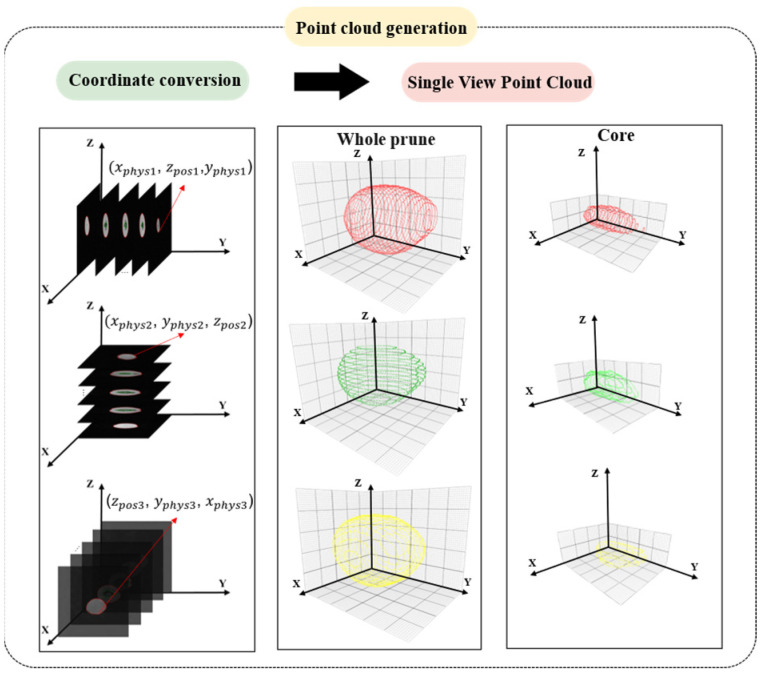
Multi-view point cloud generation from LF-NMRI images and contour extraction along three orthogonal planes.

As shown in Figure 5, the segmented slices from three orthogonal views (XY, XZ, and YZ) are mapped into 3D physical space, followed by independent contour extraction of both the whole prune and its core. The extracted boundary points are then aggregated into single-view point clouds for each imaging plane, forming the basis for subsequent registration and 3D surface modeling.

#### 2.4.2. Registration of Point Cloud Data

**Figure 6:**
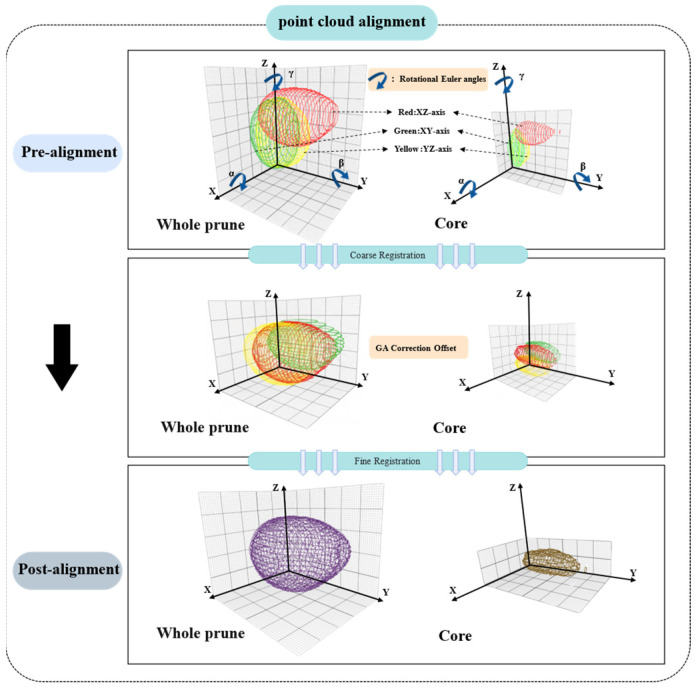
Schematic illustration of point cloud registration.

To integrate multi-view data into a unified 3D model, point clouds from the three orthogonal imaging planes must be accurately registered. The registration process comprises two sequential stages: coarse alignment and fine optimization [29]. As illustrated in Figure 6, coarse registration involves adjusting the spatial orientation of each view by rotating around Euler angles (α, β, γ), thereby achieving a preliminary morphological alignment among the point clouds and establishing the basis for precise correspondence.

Fine registration is then performed using a Genetic Algorithm (GA), a widely used evolutionary optimization technique that mimics natural selection and genetic variation [30]. Compared to traditional methods that are prone to local minima, GA offers robust global search capabilities in complex parameter spaces, making it well-suited for optimizing multi-view spatial transformations. The GA workflow consists of three key stages: population initialization, fitness evaluation, and iterative genetic operations, including selection, crossover, and mutation [31]. This structured approach ensures the effective optimization of registration parameters by simulating evolutionary processes, thereby improving the alignment accuracy of multi-view point cloud data.

In the population initialization phase, three sets of spatial point cloud data are first prepared. A population of n individuals is randomly generated to form the initial solution set P. This ensures that individuals are uniformly distributed across the predefined parameter space, enhancing the algorithm’s exploration capability and computational efficiency. One point cloud (designated as the XZ view in this study) is selected as the reference, and the central points of the other two views are translated to align with it.

The second step, fitness evaluation, quantifies the quality of registration. Since the three point clouds originate from orthogonal views of the same prune, ideal registration should result in a high degree of spatial overlap. As shown in Figure 3, the fitness of each individual is assessed based on voxel overlap between point clouds in the same spatial region, calculated as

(2) $$F(t)=V_{overlap}/(V_{overlap}+V_{independent})$$

where $V_{overlap}$ denotes the overlapping volume between two point cloud populations, calculated by multiplying the number of shared voxels by the volume of a single voxel (2.0 mm^3^). $V_{independent}$ represents the volume of non-overlapping voxels. This fitness function quantifies the proportion of spatial overlap between two sample point clouds relative to their total volume. The value of *F*(*t*) ranges from 0 to 1, with higher values indicating better registration quality.

Genetic operations constitute the final step of precision tuning, consisting of selection, crossover, and mutation. In the selection stage, a tournament selection strategy is adopted, with the tournament size set to *k* = 5. Specifically, five individuals are randomly drawn from the population to form a candidate group, and the one with the highest fitness is more likely to be selected. The probability of selection is proportional to the individual’s fitness value, as expressed by

(3) $$P(selection)\propto \max\{F(t_{1}),F(t_{2}),\ldots ,F(t_{k})\}$$

where *F*(*t_i_*) represents the fitness value of the *i*-th individual, and *P*(*selection*) is the probability of that individual being selected. This mechanism promotes the survival of high-fitness individuals through probabilistic selection, effectively guiding the population toward the optimal solution.

In the crossover step, offspring coordinates are generated by linearly interpolating the coordinates of two selected parents. Taking the x-coordinate as an example, the offspring’s coordinate is computed using

(4) $$\begin{array}{c} Child.x=random(min(P_{x1},P_{x2})-\alpha R,max(P_{x1},P_{x2})+\alpha R)\\ R=|P_{x1}-P_{x2}| \end{array}$$

where *P_x_* is the x-coordinate of a parent individual, and *R* is the absolute difference between the parent coordinates on the x-axis. The expansion factor *α* controls the exploration range of the offspring. This interpolation method enables efficient search in new solution spaces while preserving parental traits, thus improving the adaptability of offspring individuals.

Mutation introduces random variation by adding Gaussian noise to the offspring’s coordinates, calculated as

(5) $$\begin{array}{c} x^{\prime }=Child.x+N(0,\sigma^{2})\\ \sigma (t)=\sigma_{base}\cdot \left[1-(F_{avg}(t)/F_{\max}(t))\right]\;with\;\sigma_{base}=P_{m}\cdot (x_{\max}-x_{\min}) \end{array}$$

where *N* (0, *σ*^2^) denotes Gaussian noise with mean 0 and variance *σ*^2^. The standard deviation *σ(t)* is dynamically adjusted based on the fitness ratio of the average population fitness *F_avg_*(*t*) to the maximum fitness *F_max_*(*t*). The base deviation *σ_base_* is scaled by the mutation probability *P_m_* and the range of the solution space [*x*_min_, *x*_max_]. In this study, *P_m_* is set to 0.1, allowing a 10% mutation chance per individual per iteration. This mechanism promotes diversity and helps the algorithm escape local optima.

Together, the selection, crossover, and mutation operations form a complete evolutionary cycle. With successive iterations, the population gradually converges toward an optimal solution. Convergence is assessed via the fitness curve; when no further improvement is observed, the algorithm is considered to have stabilized.

The point cloud registration strategy introduced in this study is essential for multi-view data fusion, which significantly improves the accuracy and completeness of 3D surface reconstruction. In contrast, single-view modeling is often prone to detail loss and incomplete geometry, whereas multi-view fusion provides a more robust and reliable modeling framework.

#### 2.4.3. Surface Modeling of Point Cloud Data

Building upon the registered multi-view point clouds, this subsection focuses on reconstructing accurate 3D surface models of both the prune and its internal core. The Poisson surface reconstruction method [32,33] is employed, which defines the target surface as the zero isosurface of an implicit indicator function inferred from the estimated normal vector field of the point cloud.

**Figure 7:**
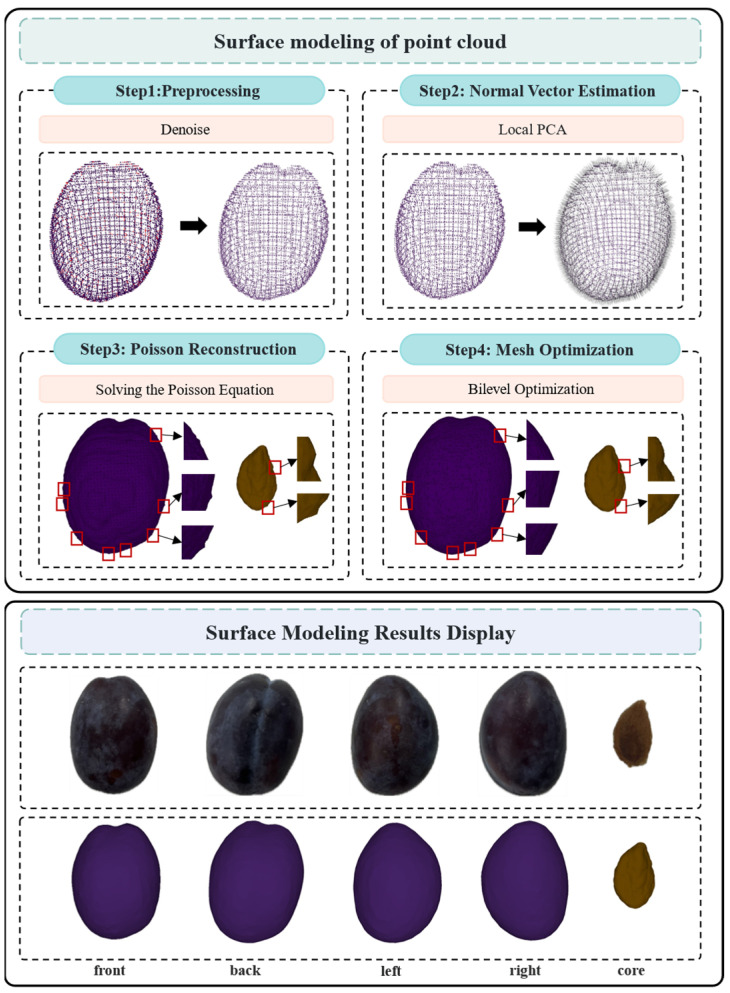
Schematic illustration of surface modeling of point cloud.

As shown in Figure 7, the reconstruction pipeline comprises four key steps. First, the fused point cloud is denoised to eliminate outliers and ensure structural coherence. Second, surface normals are estimated via Local Principal Component Analysis (LPCA), providing directional cues essential for surface fitting. Third, the Poisson equation is solved using the normal field to generate a watertight mesh that approximates the object’s geometry. Finally, a bilevel mesh optimization strategy is applied to correct local artifacts and improve overall surface smoothness. The resulting 3D models accurately capture both external and internal geometric structures of the prune. Visualizations from multiple angles, including front, back, side views and the core, highlight the method’s ability to preserve detailed morphology.

In the point cloud preprocessing stage, an adaptive voxel downsampling strategy is applied to improve data processing efficiency. For all points falling within each voxel, the centroid is calculated and used to represent the voxel. The resulting downsampled point cloud set is denoted as *P*_down_, defined by

(6) $$P_{down}=\left\{\bar{p}|\bar{p}=(1/|V_{j}|)\sum_{p_{i}\in V_{j}}p_{i}\right\}$$

where $V_{j}$ denotes the *j*-th voxel grid cell; $\left|V_{j}\right|$ is the number of points within that voxel, and $p_{i}$ is an original point in voxel $V_{j}$. The centroid $\bar{p}$ represents the average position of points in the voxel and serves as a compact representative of local geometry. This approach reduces redundancy while maintaining geometric integrity, offering computational benefits over uniform sampling [34].

To enhance the robustness of surface reconstruction, a statistical outlier removal method is applied to eliminate noise points. This approach analyzes the spatial distribution of local neighborhoods and removes outliers that deviate significantly from the average distance. Points retained by this method satisfy the condition

(7) $$P_{retained}\Leftrightarrow \|p_{i}-{\bar{p}}_{j}\|<\mu +k\sigma$$

where μ is the mean distance between a point $p_{i}$ and its neighbors, and σ is the standard deviation of neighborhood distances. The threshold parameter *k* is empirically set to 2.0. By integrating this mechanism with adaptive voxel downsampling, the geometric consistency of the point cloud is improved, providing reliable input for normal vector estimation.

Normal estimation is performed using LPCA, which extracts local surface geometry by computing the eigen decomposition of the neighborhood covariance matrix [35]:

(8) $$C=(1/|V_{j}|)\sum_{p_{i}\in V_{j}}\left(p_{i}-{\bar{p}}_{j}\right){\left(p_{i}-{\bar{p}}_{j}\right)}^{T},C=U\Lambda U^{T}$$

Here, ${\bar{p}}_{j}$ is the centroid of neighborhood $V_{j}$, and $\left|V_{j}\right|$ represents the number of in the neighborhood. The eigenvectors $u_{1}$, $u_{2}$, $u_{3}$ of matrix C describe the local surface geometry. Specifically, $u_{1}$ and $u_{2}$ span the tangent plane, while $u_{3}$ serves as the surface normal vector.

To ensure consistent orientation of normals across the point cloud, a viewpoint-based adjustment is applied:

(9) $$n_{i}=\mathrm{sgn}\left(u_{3}\cdot \left(v_{view}-p_{i}\right)\right)\cdot u_{3},N={\left\{\left.n_{i}\right\}\right.}_{i=1}^{N}$$

where $v_{view}$ is the viewpoint position and sgn(⋅) is the sign function. This ensures that all normals consistently point outward relative to the viewing direction.

Through LPCA and viewpoint consistency correction, high-precision normal vectors with consistent orientation are obtained. However, these discrete normals only describe local surface properties at sampled points and cannot directly support continuous surface reconstruction. To enable a globally differentiable surface representation, it is necessary to convert discrete normals into a continuous vector field. This is achieved by applying Gaussian kernel convolution:

(10) $$\vec{V}(x)=\sum_{i}^{N}n_{i}\cdot K_{\sigma }\left(\left\|x-p_{i}\right\|\right)$$

where $\overset{\to }{V}(x)$ denotes the continuous vector field over the surface, $K_{\sigma }(r)$ is the Gaussian kernel function, and σ is the bandwidth parameter. This formulation expresses the continuous field as a weighted sum of normal vectors, with weights determined by their proximity to *x* under the Gaussian kernel.

Based on differential geometry, a surface vector field can be viewed as a discrete sampling of the gradient of an indicator function. To reconstruct a continuous surface, a global indicator function ϕ is constructed such that its gradient field best fits the observed normal vectors. This leads to the Poisson equation:

(11) $$\nabla^{2}\phi =\nabla \vec{V}$$

where $\overset{\to }{V}$ is the smooth vector field generated from the normal vectors, ∇ is the gradient operator, and $\nabla^{2}\phi$ represents the surface’s mean curvature. Solving this equation transforms discrete normals into continuous geometric representations, enabling a topology-preserving reconstruction from point cloud to watertight mesh. The reconstructed surface corresponds to the zero level set of ϕ:

(12) $$S=\left\{\left.x|\phi (x)=0\right\}\right.$$

To improve reconstruction quality and efficiency, the Poisson reconstruction stage incorporates an octree-based multiresolution scheme and a dual-stage optimization strategy. First, Laplacian smoothing adjusts vertex positions to reduce noise and ensure uniform point distribution. Second, mesh refinement is guided by a quadratic error metric that evaluates local geometric deviations, particularly correcting surface artifacts such as protrusions around the prune and core regions (see Figure 7). Together, these steps yield smoother surfaces and more accurate geometric models while maintaining morphological details.

### 2.5. Quantitative Analysis and CVR Calculation

To quantify the 3D volumes of the core and entire fruit, this study adopts a tetrahedral summation method based on the OpenMesh library [36]. This approach enables efficient computation of tetrahedral volumes in 3D space and is well-suited for large-scale mesh processing. Based on the reconstructed mesh, the volumes of the fruit and its core are calculated separately, and the CVR is determined as

(13) $$\mathrm{CVR}=V_{\mathrm{core}}/V_{\mathrm{Whole}}$$

where *V*_core_ is the volume of the fruit core, and *V*_Whole_ is the volume of the entire fruit. This metric provides a precise quantitative indicator for evaluating internal fruit quality.

### 2.6. Evaluation Method

**Figure 8:**
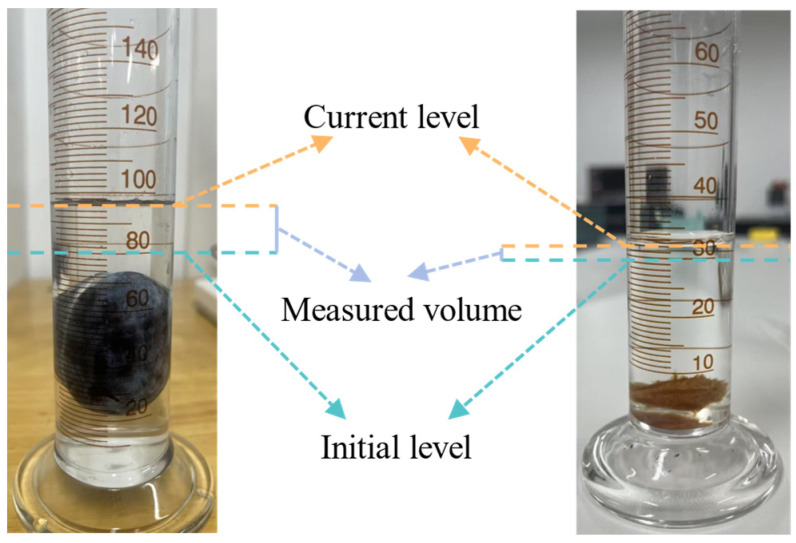
Sample prune and core volume measurement using the displacement method.

To evaluate the accuracy of the multi-view fusion 3D reconstruction, this study conducted comparative experiments using seven modeling schemes: three-view fusion (XY-XZ-YZ), dual-view combinations (XY-XZ, XZ-YZ, XY-YZ), and single-view modeling (XY, XZ, YZ). Five batches prune samples were selected, and each reconstruction scheme was applied accordingly. As shown in Figure 8, the volumes of the whole fruit and its core were measured using a water displacement method with a graduated cylinder.

The accuracy of the reconstructed volumes was then assessed by comparing them with the measured values, using mean absolute percentage error (MAPE) as quantitative indicators. The MAPE are calculated by the following equations:

(14) $$MAPE=(1/n)\sum_{i=1}^{n}\left(|M_{i}^{actual}-M_{i}^{solid}|/|M_{i}^{actual}\right|)\times 100\%$$

where $M_{i}^{solid}$ denotes the reconstructed volume and $M_{i}^{actual}$ is the measured volume.

## 3. Results and Discussion

### 3.1. Analysis of Multi-View Point Cloud Data Fusion Registration Results

**Figure 9:**
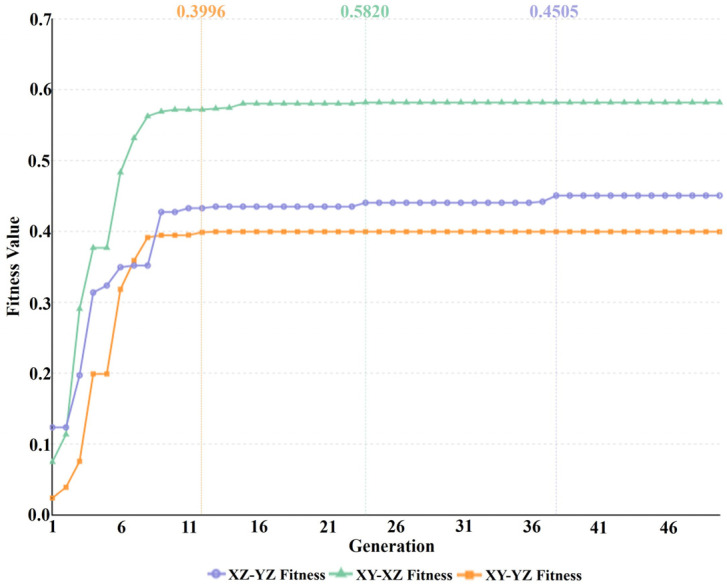
Variation curves of fitness values during multi-view point cloud fine registration for three view combinations.

The fine registration results of multi-view point clouds using the genetic algorithm are shown in Figure 9. The experiment was conducted with a population size of *n* = 50 and a maximum of 50 iterations. The fitness value, calculated based on voxel overlap (see Equation (2)), reflects the alignment quality.

As shown in the figure, all three view combinations (XZ-YZ, XY-XZ, XY-YZ) exhibit clear convergence trends. The initial fitness values range from 0.03 to 0.12, followed by rapid improvement within the first 20 generations. The final convergence levels stabilize around 0.45 (XZ-YZ), 0.58 (XY-XZ), and 0.40 (XY-YZ). These results indicate that the registration strategy effectively corrects spatial misalignments across views, enabling consistent geometric fusion.

### 3.2. Multi-View 3D Reconstruction Result Analysis

**Figure 10:**
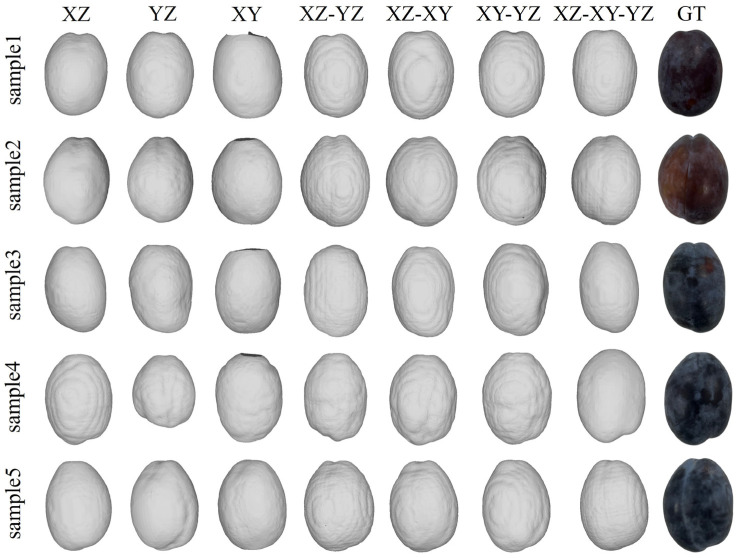
Comparative visualization of 3D reconstruction results for five entire fruit samples randomly selected from five batches under different modeling schemes.

The 3D reconstruction results for examples from the five batches of prune samples under seven schemes are presented in Figure 10. Among them, models reconstructed from single-view inputs are the roughest, with obvious staircase artifacts caused by sparse slice sampling. In particular, the XY view produces local voids near the top and bottom regions due to insufficient data density. Dual-view combinations alleviate surface artifacts to some extent, yielding smoother geometry and improved detail continuity, though minor defects persist.

**Figure 11:**
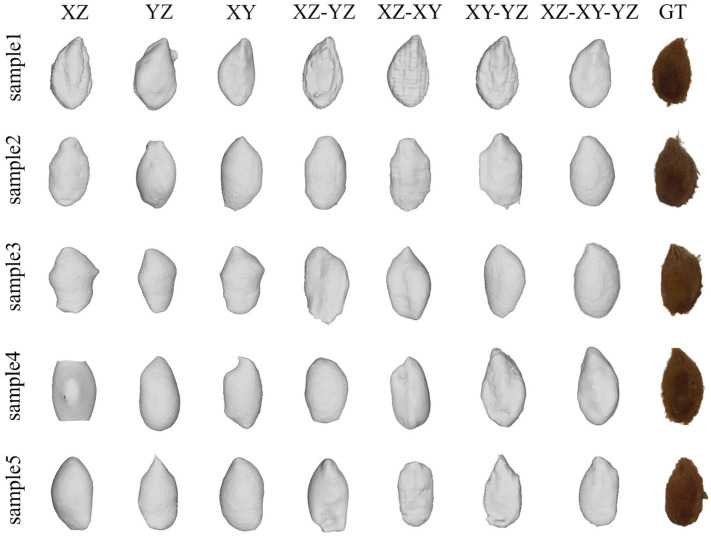
Comparative visualization of 3D reconstruction results for five core samples randomly selected from five batches under different modeling schemes.

In contrast, the proposed three-view approach (XZ-XY-YZ) shows substantial improvements, generating visually continuous surfaces and accurately reconstructing biological features such as longitudinal grooves. As shown in Figure 10, it achieves high global and local fidelity, benefiting from a dual-layer optimization strategy that balances surface smoothness with structural precision. This advantage extends to internal core modeling: Figure 11 demonstrates that the three-view scheme generates core models that most closely resemble the ground truth, while single-view and dual-view approaches suffer from visible deformation or artifact retention. These results confirm the method’s superior capability to recover both internal and external structures with high morphological fidelity.

Quantitative analysis based on the five production batches (Table 1) demonstrates a clear hierarchy in reconstruction accuracy and stability among the modeling schemes. The three-view fusion strategy (XZ-XY-YZ) achieves the highest overall accuracy, with the MAPE of 0.73% and the smallest standard deviation (±0.17%), indicating superior robustness against inter-fruit morphological variations. In contrast, single-view schemes show higher and more variable errors, with the XY view performing the worst (MAPE: 7.68%, SD: ±1.66%), a result attributable to its acquisition of the fewest image slices. Dual-view integrations reduce the mean error compared to single views but do not match the precision and consistency of the three-view approach.

The overall performance obtained in this study falls within a similarly low error range to that reported in previous LF-NMRI-based studies [23]. Wang et al. reported that PVR estimation errors were below 1%, and below 0.5% when using the integral method. Although the average error obtained in the present study (MAPE = 0.73%) is slightly higher than the best value reported in that work, the previous study was validated using only three fruits, which limits the statistical representativeness of that comparison. In contrast, the present study evaluates the proposed method across five production batches and reports both mean error and standard deviation, thereby providing a more statistically informative assessment of reconstruction accuracy and stability.

**Figure 12:**
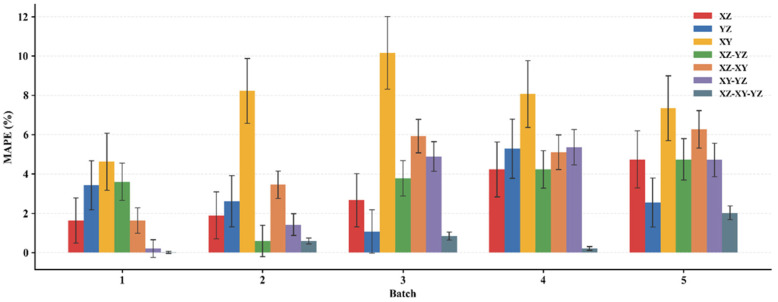
Comparison of reconstruction accuracy (MAPE) for different modeling schemes across five prune batches.

The consistency of this performance advantage is visually affirmed across all individual batches, as detailed in Figure 12. The bar chart illustrates that the three-view fusion scheme not only yields the lowest error bar in each batch but also exhibits the smallest fluctuation in MAPE values from batch to batch. This visual evidence highlights its decisive role in minimizing outlier variance and ensuring reliable performance despite natural sample variations. While dual-view combinations reduce the mean error relative to single views, the integration of the third orthogonal view is critical for achieving both the lowest error and the highest stability, effectively mitigating structural distortions inherent in limited-view reconstructions.

Together, the visual and quantitative results demonstrate that the proposed multi-view fusion approach significantly enhances 3D reconstruction quality by reducing artifacts, preserving morphological details, and improving volumetric accuracy. These improvements collectively validate the effectiveness and robustness of the proposed method.

**Figure 13:**
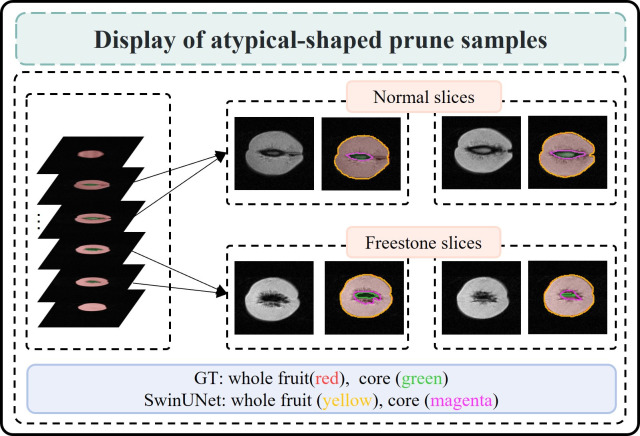
Representative slices from the same slice set of atypical prune samples, illustrating both clear and ambiguous core–flesh segmentation boundaries.

Notably, the fifth batch exhibited relatively larger errors across multiple reconstruction schemes. To further examine this observation, representative prune slices with clear and ambiguous core–flesh boundaries are presented in Figure 13. As shown in the figure, some samples displayed less distinct internal boundaries and more complex morphological characteristics, which may increase the difficulty of accurate reconstruction. In addition, because the multi-view reconstruction pipeline relies on spatial alignment across different views, potential registration inaccuracies may also contribute to the final reconstruction error. These cases can be regarded as borderline or failure-prone examples, in which atypical morphology and ambiguous boundaries make accurate segmentation and reconstruction more challenging.

Nevertheless, the three-view fusion scheme still achieved the lowest error in this batch, further demonstrating its robustness under relatively challenging cases. This superior performance can be attributed to the complementary geometric information provided by the third orthogonal view, which cannot be sufficiently recovered from single-view or dual-view inputs alone. Under sparse LF-NMRI sampling conditions, missing information along one direction can propagate into local shape errors during surface reconstruction, whereas multi-view fusion alleviates this problem by constraining the reconstructed geometry from multiple orthogonal perspectives, thereby improving structural completeness and reducing reconstruction distortion.

### 3.3. CVR Calculation and Analysis

Based on the high-precision 3D reconstruction models, both the external morphology and internal core structures of the prunes were quantified at the millimeter scale, providing a solid foundation for internal morphological analysis. The CVR is a key indicator of fruit quality, as it directly reflects the edible portion of the fruit.

Using the three-view (XZ-YZ-XY) reconstruction method, the CVR of five samples was estimated and validated against physical measurements. The reconstructed core volumes were compared with physical measurements obtained via water displacement using a graduated cylinder (Table 2). The reconstructed core volumes closely matched the measured values, with a Mean Absolute Error (MAE) of only 0.13%, and an error range from −0.26% to +0.39%, demonstrating high accuracy and reproducibility.

CVRs were then calculated based on both the modeled and measured core volumes. Although the MAE in CVR estimation remained low at just 0.13%, the MAPE reached 4.90%, which is notably higher than the corresponding volume estimation error. This is mainly due to the small size of the core, where slight deviations in volume lead to amplified CVR errors. Additionally, the uncertainty of the graduated cylinder used for volume measurement likely exceeded the actual modeling error, further contributing to the elevated MAPE. Nevertheless, the modeled CVR values remained in close agreement with manual measurements, demonstrating the reliability and precision of the proposed multi-view reconstruction approach. These results indicate that the proposed framework is not only suitable for morphological visualization, but also reliable for quantitative trait extraction. This is particularly relevant for CVR measurement, where even small reconstruction bias in the core can directly affect the final quality evaluation.

**Figure 14:**
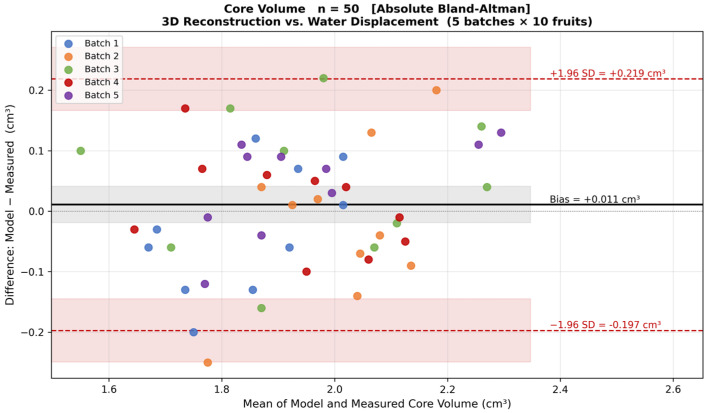
Bland–Altman analysis of core volume agreement between 3D reconstruction and water displacement measurement across 50 prune samples (5 batches × 10 fruits).

To further evaluate the agreement between the 3D reconstruction and the water displacement method, a Bland–Altman analysis was performed on the core volume measurements across all 50 prune samples. As shown in Figure 14, the black solid line is the mean difference, and the red dashed lines are the 95% limits of agreement. Most data points fall within these limits, indicating good consistency between the two methods.

From a biological perspective, the CVR of prune reflects the relative volumetric relationship between the endocarp and the whole fruit, and its variation is closely related to differences in the growth rates of the core and pulp at different developmental stages. During the young fruit stage, both the core and the pulp are still growing, and CVR remains dynamic. During the fruit expansion stage, the increase in fruit volume is mainly driven by the rapid enlargement of the mesocarp, whereas endocarp development gradually approaches completion and becomes relatively stable; therefore, CVR tends to decrease [37]. As the fruit approaches maturity, the core volume changes little, and CVR generally becomes relatively stable. This pattern is consistent with the general framework of fleshy fruit development and ripening [38].

However, during the postharvest stage, even though the core itself remains largely unchanged, ripening after harvest may still induce pulp softening through cell wall polysaccharide disassembly and microstructural modifications [39]. These tissue changes may not only affect the biological interpretation of CVR, but also reduce the clarity of the boundary between the core and the pulp in LF-NMRI images, thereby becoming an important source of segmentation uncertainty. Therefore, the proposed non-destructive method may provide a valuable technical basis for future longitudinal studies on internal structural changes in stone fruits during ripening and storage.

## 4. Conclusions

This study presents a high-precision 3D quantitative method for characterizing the quality of stone fruits using LF-NMRI. Tomographic image sequences acquired along three orthogonal axes were converted into multi-view point cloud data, which were registered using a genetic algorithm and fused via Poisson surface reconstruction to generate a detailed 3D model. Applied to prunes, the method accurately reconstructs both the external morphology and internal core, allowing precise measurement of the CVR, a critical indicator of stone fruit quality. The three-view reconstruction significantly improved modeling accuracy, reducing the MAPE to 0.73%, compared to 4.57% and 3.73% for single-view and dual-view methods, respectively. Core volume estimation yielded an MAE of 0.13%, with deviations ranging from −0.26% to +0.39%, demonstrating excellent accuracy and reproducibility.

However, the efficiency of the current framework remains constrained by limitations at both the acquisition and computation levels, namely the time-consuming multi-view sampling required by LF-NMRI and the additional computational cost associated with deep learning-based segmentation. Enhancing sampling speed while preserving accuracy will be a critical direction for future research. In addition, further validation on larger and more diverse datasets and across additional fruit types will be necessary to better establish the robustness and generalizability of the proposed method.

## Figures and Tables

**Table 1:** Volumetric measurement statistics and reconstruction error distribution for five prune batches under different multi-view fusion schemes.

Batch	Mean (cm^3^)	Single-View Error (%)			Dual-View Error (%)			Three-View Error (%)
		XZ	YZ	XY	XZ-YZ	XZ-XY	XY-YZ	XZ-XY-YZ
1	28.00	[0.48, 2.78]	[2.17, 4.67]	[3.17, 6.07]	[2.65, 4.55]	[0.98, 2.28]	[−0.25, 0.65]	[ − 0.04, 0.06]
2	36.00	[0.69, 3.09]	[1.31, 3.91]	[6.57, 9.87]	[−0.21, 1.39]	[2.75, 4.15]	[0.87, 1.97]	[0.44, 0.74]
3	34.00	[1.31, 4.01]	[−0.03, 2.17]	[8.31, 12.01]	[2.88, 4.68]	[5.07, 6.77]	[4.14, 5.64]	[0.64, 1.04]
4	35.00	[2.83, 5.63]	[3.78, 6.78]	[6.36, 9.76]	[3.28, 5.18]	[4.22, 5.98]	[4.46, 6.26]	[0.11, 0.31]
5	34.00	[3.29, 6.19]	[1.29, 3.79]	[5.69, 8.99]	[3.69, 5.79]	[5.32, 7.22]	[3.86, 5.56]	[1.67, 2.37]
MAPE ± SD		3.03 ± 1.31	2.98 ± 1.28	7.68 ± 1.66	3.39 ± 0.93	4.47 ± 0.81	3.32 ± 0.70	0.73 ± 0.17

**Table 2:** Comparison of modeled and measured core volumes and CVRs for five prune samples based on three-view reconstruction (cm^3^).

Sample	Model Core Volume	Measured Value	Model CVR	Measured CVR	Error	MAPE
1	1.97	1.90	7.04%	6.78%	+0.26%	3.83%
2	2.13	2.00	5.95%	5.56%	+0.39%	7.01%
3	2.04	2.10	6.06%	6.18%	−0.12%	1.94%
4	1.90	2.00	5.45%	5.71%	−0.26%	4.55%
5	1.89	1.80	5.67%	5.29%	+0.38%	7.18%
Mean	1.99	1.96	6.03%	5.90%	+0.13%	4.90%

## Data Availability

The original contributions presented in the study are included in the article, further inquiries can be directed to the corresponding author.
